# Integrated bioinformatics combined with machine learning to analyze shared biomarkers and pathways in psoriasis and cervical squamous cell carcinoma

**DOI:** 10.3389/fimmu.2024.1351908

**Published:** 2024-05-28

**Authors:** Luyu Liu, Pan Yin, Ruida Yang, Guanfei Zhang, Cong Wu, Yan Zheng, Shaobo Wu, Meng Liu

**Affiliations:** ^1^ Department of Dermatology, The First Affiliated Hospital of Xi’an Jiaotong University, Xi’an, Shaanxi, China; ^2^ Department of Medicine, Xi’an Jiaotong University, Xi’an, Shaanxi, China

**Keywords:** psoriasis, cervical squamous cell carcinoma (CESC), immune cell infiltration, machine learning, biomarkers

## Abstract

**Background:**

Psoriasis extends beyond its dermatological inflammatory manifestations, encompassing systemic inflammation. Existing studies have indicated a potential risk of cervical cancer among patients with psoriasis, suggesting a potential mechanism of co-morbidity. This study aims to explore the key genes, pathways, and immune cells that may link psoriasis and cervical squamous cell carcinoma (CESC).

**Methods:**

The cervical squamous cell carcinoma dataset (GSE63514) was downloaded from the Gene Expression Omnibus (GEO). Two psoriasis-related datasets (GSE13355 and GSE14905) were merged into one comprehensive dataset after removing batch effects. Differentially expressed genes were identified using Limma and co-expression network analysis (WGCNA), and machine learning random forest algorithm (RF) was used to screen the hub genes. We analyzed relevant gene enrichment pathways using GO and KEGG, and immune cell infiltration in psoriasis and CESC samples using CIBERSORT. The miRNA-mRNA and TFs-mRNA regulatory networks were then constructed using Cytoscape, and the biomarkers for psoriasis and CESC were determined. Potential drug targets were obtained from the cMAP database, and biomarker expression levels in hela and psoriatic cell models were quantified by RT-qPCR.

**Results:**

In this study, we identified 27 key genes associated with psoriasis and cervical squamous cell carcinoma. NCAPH, UHRF1, CDCA2, CENPN and MELK were identified as hub genes using the Random Forest machine learning algorithm. Chromosome mitotic region segregation, nucleotide binding and DNA methylation are the major enrichment pathways for common DEGs in the mitotic cell cycle. Then we analyzed immune cell infiltration in psoriasis and cervical squamous cell carcinoma samples using CIBERSORT. Meanwhile, we used the cMAP database to identify ten small molecule compounds that interact with the central gene as drug candidates for treatment. By analyzing miRNA-mRNA and TFs-mRNA regulatory networks, we identified three miRNAs and nine transcription factors closely associated with five key genes and validated their expression in external validation datasets and clinical samples. Finally, we examined the diagnostic effects with ROC curves, and performed experimental validation in hela and psoriatic cell models.

**Conclusions:**

We identified five biomarkers, *NCAPH, UHRF1, CDCA2, CENPN*, and *MELK*, which may play important roles in the common pathogenesis of psoriasis and cervical squamous cell carcinoma, furthermore predict potential therapeutic agents. These findings open up new perspectives for the diagnosis and treatment of psoriasis and squamous cell carcinoma of the cervix.

## Introduction

Psoriasis is a chronic inflammatory and hyperproliferative skin condition, which is mediated by the immune system. The inflammatory features have been acknowledged with a deeper understanding of its biological properties (1–6). Several co-morbidities such as metabolic syndrome, tumors and inflammatory diseases can be induced by the cytokines involved in psoriasis (7–12). In addition, psoriasis patients receiving systemic and UV therapy are more likely to develop general and organ-specific cancers (13, 14).

Cervical cancer is a malignant tumor that arises in the cervix and vagina, with the second highest incidence rate among female tumors (15). Furthermore, it remains the second most common cause of cancer-related deaths among women in developing nations (16). The incidence of cervical cancer is on the rise, necessitating further exploration of new treatments for cervical squamous cell carcinoma (17, 18). The grave issue of patients with advanced cervical cancer experiencing poor prognosis and survival rates persists (19, 20). Previous studies have shown that the pathogenesis of cervical cancer is hypothesized to stem from multifactorial interactions between the host system, HPV(Human Papilloma Virus) infection, and diverse behavioral, environmental, or inherited variables (21).

Clinical data reveals that the majority of patients presenting with both cervical cancer and psoriasis exhibit advanced inoperable stages or postoperative recurrence. These cases are characterized by pathologically confirmed squamous cell carcinoma, a history of psoriasis, and a recurrent pattern of immunosuppressive therapy usage (22, 23). A traditional Chinese medicine known as Wolf Poison demonstrates dual efficacy—internally for treating cervical cancer and externally for addressing psoriasis. This dual therapeutic application suggests a potential common pathogenesis between cervical cancer and psoriasis (24, 25). In addition, both psoriasis and cervical squamous cell carcinoma show hyperproliferation of squamous epithelial cells and both have angiogenic mechanisms (26–29). Several studies have suggested that prolonged immunosuppression in individuals with psoriasis hampers immune responses, elevating their vulnerability to tumorigenesis, including CESC (30–32). However, the underlying mechanisms of this comorbidity remain unclear and warrant further investigation.

Thus, this study employs a systems biology approach to elucidate potential biomolecular mechanisms shared between psoriasis and CESC. Our findings aim to identify candidate biomarker signatures that could be common between psoriasis and cervical squamous cell carcinoma, contributing valuable insights to the field.

## Materials and methods

### Data processing

**Figure 1:**
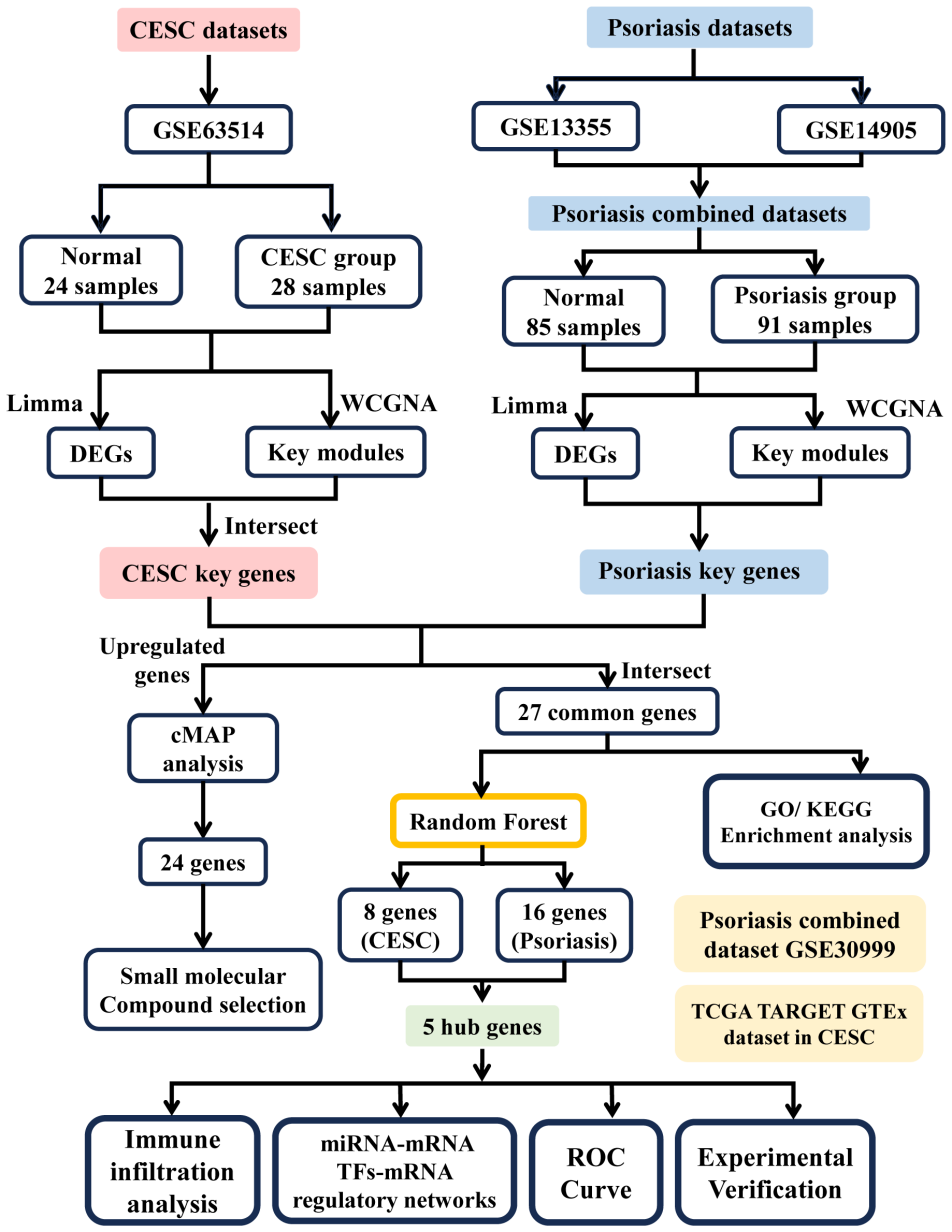
Flowchart of analytical steps in this study.

The research flowchart of this research is shown in **Figure 1**. Data Source GEO (http://www.ncbi.nlm.nih.gov/geo) is a public database containing a large number of high-throughput sequencing and microarray datasets submitted by research organizations around the world. The epithelial cell microarray dataset of cervical squamous cell carcinoma patients (GSE63514), including 24 normal 28 cervical squamous cell carcinoma epithelial cell specimens, was obtained through GEO. Two expression profiling datasets, GSE13355 and GSE14905, were downloaded from the GEO database for psoriasis and controls. The GSE13355 dataset consisted of total RNA extracted from puncture biopsies of 58 patients with psoriasis and 64 normal healthy controls, and the GSE14905 dataset consisted of skin biopsy specimens from 21 normal healthy donors and 56 from 28 patients with psoriasis skin biopsy samples. Batch correction integration, normalization, and gene ID transformation were performed on the 2 psoriasis datasets carried out using the R software package SVA (v4.2.1). RNAseq data for the STAR process of the TCGA-CESC project were downloaded and organized from The Cancer GenomeAtlas Program (TCGA) database (https://portal.gdc.cancer.gov) and extracted in TPM format. **Table 1** presents detailed dataset information, including the microarray platform, sample groups, and numbers.

**Table 1:** Basic information of datasets used in the study.

Datasets	Type	Sample size		Platform
		Normal	Psoriasis	
**GSE13355**	RNA	64	58	GPL570
**GSE14905**	RNA	21	33	GPL570
**GSE30999**	RNA	85	170	GPL570
		Control	CESC	
**GSE63514**	RNA	24	28	GPL570
**TCGA-CESC**	RNA	3	306	

### Identification of DEGs

Limma, a differential expression screening method based on generalized linear models, was utilized to obtain the differential genes between different comparator groups and the control group. We conducted the differential analysis using the R package limma (version 3.40.6) (33). We obtained the expression profiling dataset and performed multiple linear regression utilizing the lmFit function. We then utilized the eBays function to compute moderated t-statistics, moderated F-statistics, and log-odds of differential expression through empirical Bayes moderation of the standard errors towards a common value. Finally, we determined the significance of differences for each gene. Technical terms were explained upon first usage and the language used was neutral and objective.

### Weighted gene co-expression network analysis

Using gene expression profiles, we calculated the MAD (Median Absolute Deviation) of each gene separately, eliminated the top 50% of genes with the smallest MAD, removed outlier genes and samples using the goodSamplesGenes method of the R package WGCNA, and further constructed scale-free co-expression networks using WGCNA. β is a soft-threshold parameter that can emphasize strong correlations between genes and penalize weak correlations. The neighbor-joining matrix was converted to a topological overlap matrix (TOM), which measures the network connectivity of a gene, defined as the sum of the neighbor-joining matrices of the gene and all other genes assigned to the network gene, and the corresponding dissimilarity (1-TOM) was calculated. To cluster genes with similar expression profiles into gene modules, we utilized average linkage hierarchical clustering based on the TOM similarity measure. It should be noted that the gray modules were classified as the set of genes unassigned to any module.

### PPI network construction and module analysis

Search Tool for the Retrieval of Interacting Genes (STRING, http://string-db.org) (version 11.0) searches for relationships between proteins of interest, such as direct binding relationships, or coexisting upstream and downstream regulatory pathways, to construct PPI networks with complex regulatory relationships.

### Functional enrichment analysis

Sangerbox (http://www.sangerbox.com/tool) was used for Gene Ontology (GO) and Kyoto Encyclopedia of Genes and Genomes (KEGG) enrichment analysis. Gene Ontology (GO) analysis is a common technique utilized for conducting large-scale functional enrichment studies that encompass biological processes, molecular functions, and cellular components (34). Kyoto Encyclopedia of Genes and Genomes (KEGG) is a popular database for storing information pertaining to genomes, biological pathways, diseases, and pharmaceuticals (35). Adjusted P-value < 0.05 was considered significant.

### Machine learning

Machine learning algorithms are used to screen the core genes for diagnosis. Using the Random Forest (RF) algorithm which integrates multiple trees for better accuracy through the idea of ensemble learning, we narrowed down the candidate biomarkers, which integrates multiple trees for better accuracy through the idea of ensemble learning. The genes with MeanDecreaseGini > 2 in the RF model were defined as the central genes.

### Immune infiltration analysis

The CIBERSORT algorithm is utilized for evaluating the percentage of immune cells present in cells or tissues. The bar graphs show the proportion of each type of immune cell in various samples, and the “corrplot” R package is used to generate a heat map of the correlation between 22 immune cells. The vioplot was used to visualize the differences between the Psoriasis and normal immune cell groups.

### Identification of transcription factors and miRNAs interact with key genes

Hub transcription factors (TFs) were identified using the JASPAR database, and the effect of binding of hub miRNAs to hub gene transcripts on protein expression was detected by miRNet (https://www.mirnet.ca/). We constructed topological networks of TFs genes and miRNA genes using Cytoscape software.

### Isolation of human primary keratinocytes

Skin samples were obtained from the foreskin tissue of eight children, aged 6 to 12 years, at Northwest Women’s and Children’s Hospital in Xi’an, China. Prior to the procedure, the researchers obtained ethical permits and secured written informed consent from the parents or legal guardians of the participants. The researchers isolated primary keratinocytes using the standard two-step digestion method (36).

### Cell culture

The HeLa cells were acquired from ATCC and grew in DMEM with 10% fetal bovine serum, penicillin (100 U/mL), and streptomycin (100 μg/mL) at 37°C in a humidified atmosphere with 5% CO2. PKC was cultured following prior procedures (37).

### Establishment of the psoriatic cell model

PKCs were stimulated by M5 (TNF-a, IL-17A, IL-22, IL-1a, and oncostatin M) at a concentration of 10 ng/mL for a duration of 24 hours, as previously described (37).

### qRT-PCR

RNA extraction and qRT-PCR procedures were conducted following the previously described method (38). Relevant mRNA levels were determined utilizing the 2^^(-ΔΔCt)^ formula. The primers used in the study are summarized in **Table 2**.

**Table 2:** Basic information of datasets used in the study.

Gene	Forward (5’→3’)	Reverse (5’→3’)
***CDCA2***	TCTGATTCGTTTCATTGCTCGG	ACATTTCGATACAGTGCAGGG
***CENPN***	TGAACTGACAACAATCCTGAAGG	CTTGCACGCTTTTCCTCACAC
***MELK***	AACTCCAGCCTTATGCAGAAC	AACGATTTGGCGTAGTGAGTATT
***NCAPH***	GTCCTCGAAGACTTTCCTCAGA	TGAAATGTCAATACTCCTGCTGG
***UHRF1***	AGGTGGTCATGCTCAACTACA	CACGTTGGCGTAGAGTTCCC

### Statistical analysis

All statistical analyses were conducted using R software version 4.2.2 and Sangerbox. To assess the statistical significance between normally distributed variables in the two groups of continuous variables, we employed the independent Student’s t-test. Conversely, differences between non-normally distributed variables were determined using the Mann-Whitney U-test. The statistical significance between the two groups of categorical variables was analyzed using either the chi-square test or Fisher’s exact test. Estimation of correlation coefficients between different genes was conducted through Pearson correlation analysis. All statistical tests conducted were two-sided and the level of statistical significance was set at a p-value of less than 0.05.

## Results

### WGCNA identifies key modules in psoriasis and cervical cancer

**Figure 2:**
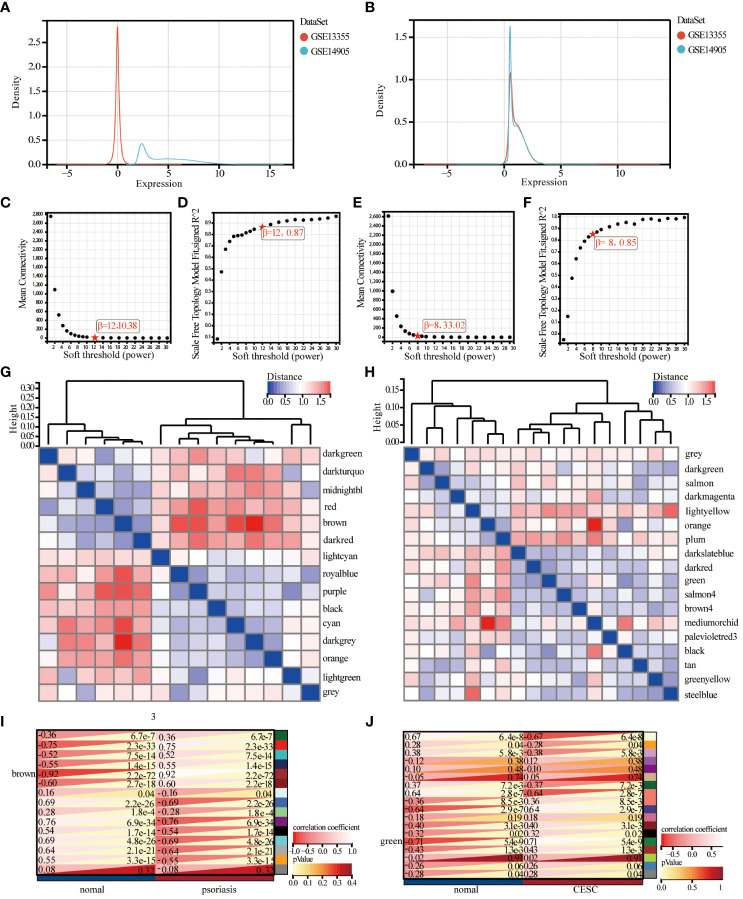
Identification and analysis of key module of psoriasis and cervical squamous cell carcinoma by WGCNA. **(A)** Principal component analysis of the two original Psoriasis datasets before batch effect correction. **(B)** Principal component analysis of the corrected Psoriasis dataset. **(C, D)** Scale independence and average connectivity plots of psoriasis. **(E, F)** Scale independence and average connectivity plots of cervical cancer. **(G, H)** Gene dendrogram and heatmap of the modular signature gene network. **(I, J)** Identification of weighted gene co-expression network modules associated with psoriasis and cervical cancer, and module characterized genes in relation to psoriasis and cervical cancer status.

The investigators merged two psoriasis-related GEO datasets, GSE14905 and GSE13355. The data sets were merged and normalized to ensure uniformity for principal component analysis and to rectify batch effects. The final training dataset consisted of 91 patients and 85 matched controls, and the evaluation showed that the data preprocessing was valid and reliable. From the density plot, we can observe that the sample distributions of the individual datasets before removing the batch effect varied greatly, suggesting a batch effect, and after removing the batch effect the data distributions between the individual datasets converged, with similar means and variances (**Figures 2A, B**). Weighted gene co-expression network analysis was conducted utilizing the R package WGCNA, the genes with expression variance in the top 50% were used as the screening conditions, and the genes with less volatility were excluded, and the co-expression network was constructed for 20547 genes of psoriasis and 10,275 genes of cervical cancer.

Combining the analysis of scale independence and average connectivity, in the psoriasis samples, b=12 was chosen (**Figures 2C, D**) as the soft threshold. The minimum module size was set to 30 and 15 gene modules were obtained (**Figure 2G**). The results showed that the brown module had the highest correlation with psoriasis (correlation coefficient = 0.92, p= 2.2e-72, **Figure 2I**). Ultimately, 969 psoriasis-significantly correlated genes were identified in brown color module with high MM (> 0.8) and GS (> 0.1) values. In the cervical cancer samples, 8 was chosen as the optimal soft threshold to build a scale-free network (**Figures 2E, F**). Subsequently, cluster analysis was used to identify highly similar modules with the minimum module size set to 30, sensitivity set to 3, and modules with distances less than 0.25 were merged to obtain 18 gene modules (**Figure 2H**). The correlation between cervical cancer and gene modules (**Figure 2J**) showed that the green module had the highest correlation with cervical cancer (2270 genes, r=0.71, p=5.4e-9), and the green module was taken as the key module. The genes in the green module: MM>0.8 and GS>0.1 were selected as pivotal genes, and a total of 421 key genes significantly associated with cervical cancer were identified.

### Identification of differentially expressed genes and machine learning screening of key genes

**Figure 3:**
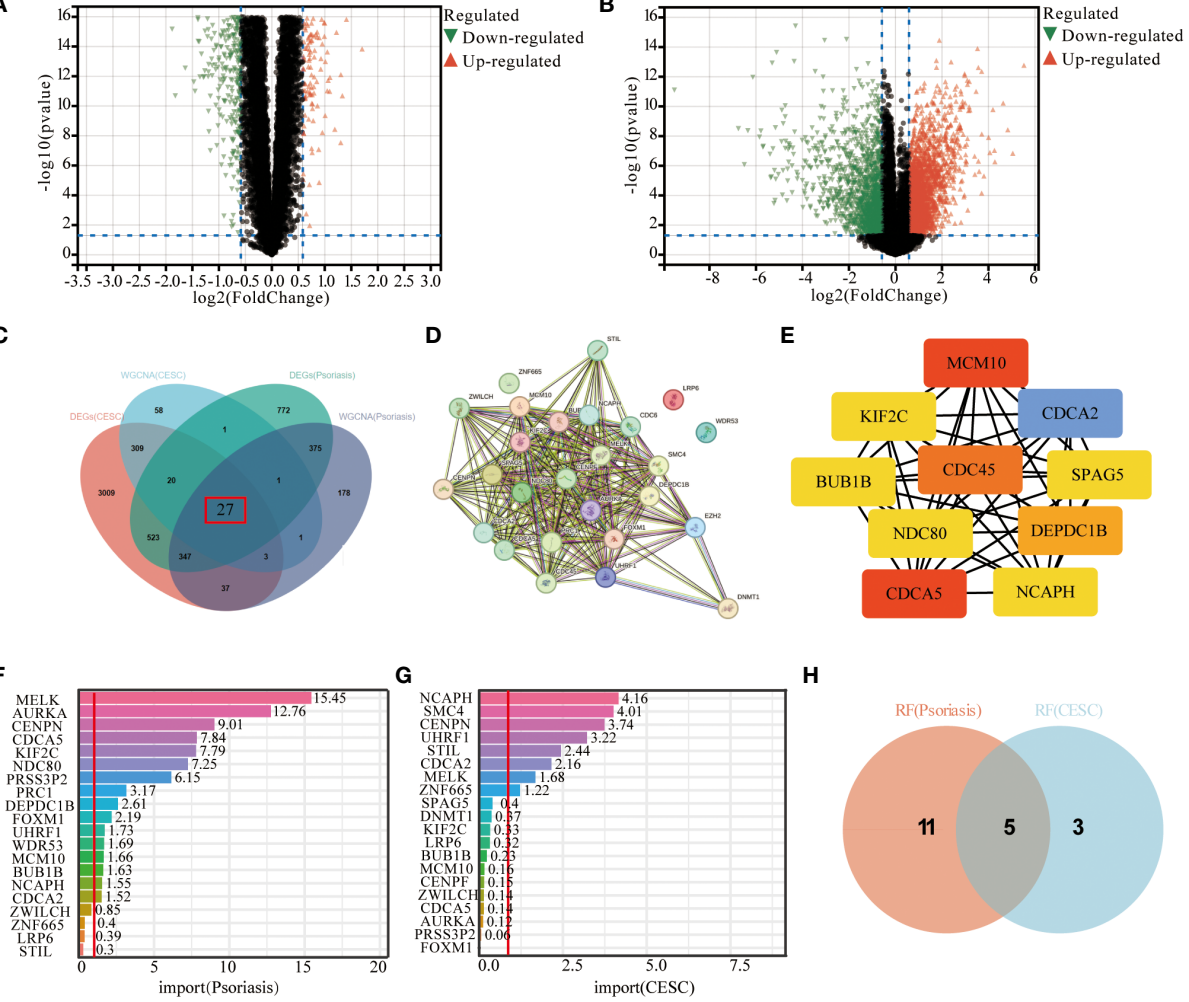
Screening of hub-genes by machine learning algorithm. **(A, B)** Volcano plot demonstrating an overview of the differential expression of all genes in CESC and Psoriasis. **(C)** DEGs in cervical cancer and psoriasis samples were intersected with key genes in WGCNA taken to obtain the Wayne plots of 27 genes. **(D)** PPI network of 27 genes. **(E)** Major PPI network analysis of the top 10 hub genes by CytoHubba software. **(F)** RF algorithm screened out 16 characterized genes in psoriasis samples. **(G)** The RF algorithm screened 8 characterized genes in cervical cancer samples. **(H)** Wayne diagram of 5 key genes identified. The threshold in the volcano plot was -log10 (adjusted P-value) > 2 and |log2 (fold change)| > 0.5; red dots indicate significant differential expressed genes. FDR was used for P value adjustment.

By Limma analysis, 2066 differentially expressed genes (DEGs) between psoriasis patients and healthy controls were identified in the integrated dataset, of which 1134 genes were up-regulated and 932 genes were down-regulated. These DEGs were presented by volcano plot visualization (**Figure 3A**). In addition, the cervical squamous cell carcinoma dataset generated 6573 DEGs, including 2689 up-regulated genes and 1586 down-regulated genes (**Figure 3B**). The DEGs from the cervical squamous cell carcinoma and psoriasis samples were intersected with key genes taken from the WGCNA to obtain a total of 27 genes for subsequent analysis (**Figure 3C**). 27 genes were uploaded to the STRING database to construct a protein-protein interaction network (**Figure 3D**), then we analyzed the top 10 genes by using the “degree” algorithm with the CytoHubba application in Cytoscape to identify the key genes, and the color of the nodes indicated the strength of the correlation (**Figure 3E**). The color of the nodes indicates the strength of the correlation. Random forest pairs were used for screening and finally 16 characterized genes were identified in psoriasis samples, including *MELK*, *AURKA*, *CENPN*, *CDCA5*, *KIF2C*, *NDC80*, *PRSS3P2*, *PRC1*, *DEPDC1B*, *FOXM1*, *UHRF1*, *WDR53*, *MCM10*, *BUB1B*, *NCAPH*, *CDCA2*(**Figure 3F**). Meanwhile, 10 cervical squamous cell carcinoma signature genes were also identified using RF algorithms, including *NCAPH, SMC4, CENPN, UHRF1, STIL, CDCA2, MELK, ZNF665* (**Figure 3G**). Next, the study found that these algorithms identified five overlapping genes (**Figure 3H**), namely *NCAPH, UHRF1, CENPN, CDCA2, MELK* which were used for sebsequent analysis (**Table 3**).

**Table 3:** Overview of the five hub genes.

Symbol	Description	Aspect	References
*NCAPH*	Non-SMC Condensin I Complex Subunit H	Interferes with plasmids and affects cell proliferation and migration	(39)
*UHRF1*	Ubiquitin Like With PHD And Ring Finger Domains 1	Required for G1/S phase transition; Regulation of DNA methylation, chromatin modification, cell proliferation and DNA repair	(40, 41)
*CENPN*	Centromere Protein N	Binds to filaments in S and G2 phases and recruits proteins	(42)
*CDCA2*	Cell Division Cycle Associated 2	Affects tumor cell proliferation and regulates the G0/G1 phase of the cell cycle	(43)
*MELK*	Maternal Embryonic Leucine Zipper Kinase	Induces inflammatory responses through secretion of pro-inflammatory factors Involved in mitosis, proliferation, apoptosis, differentiation and tumorigenesis	(44, 45)

### GO and KEGG enrichment analyses were performed to identify biological pathways and diseases associated with key genes

**Figure 4:**
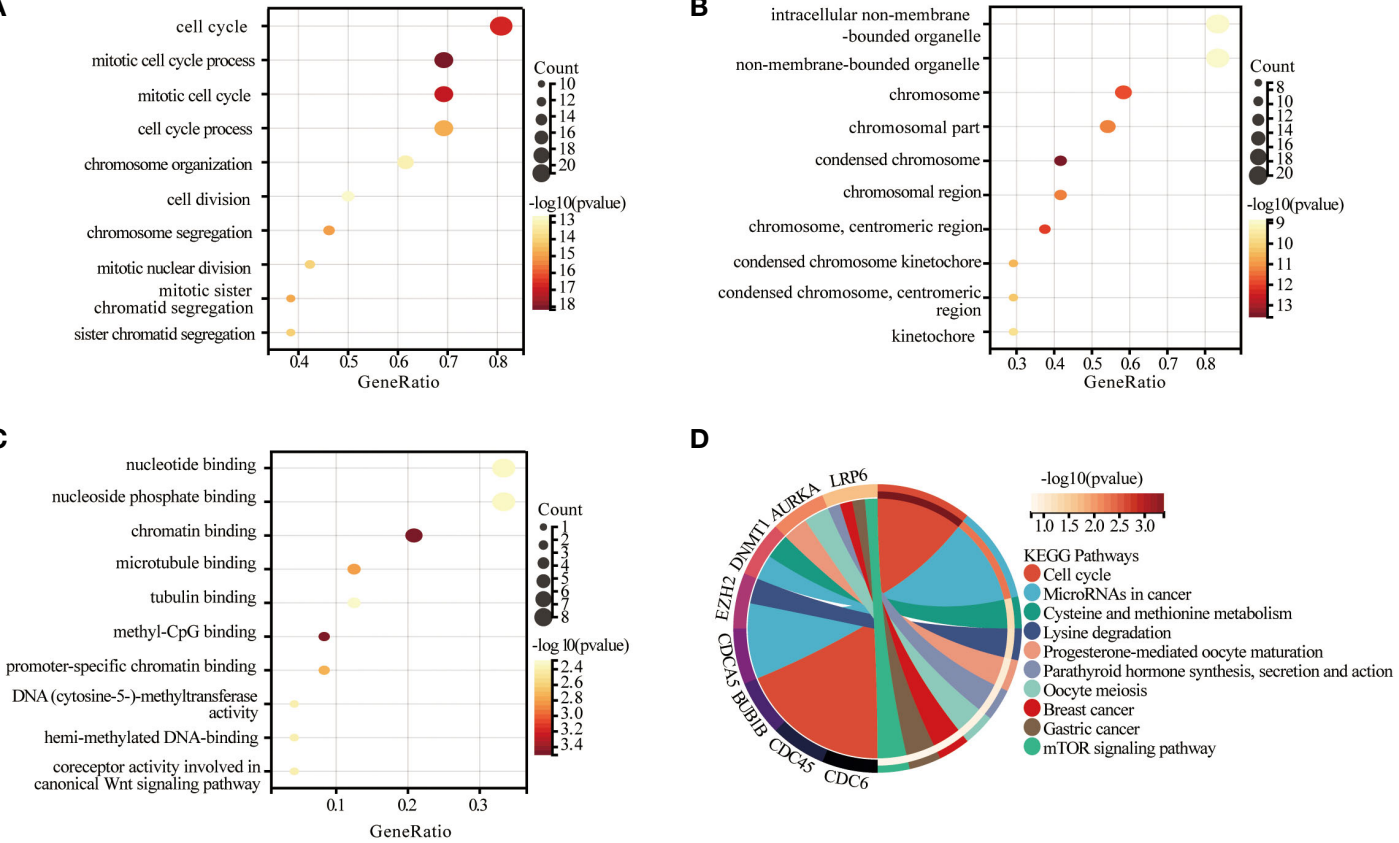
Significant gene module and enrichment analysis of the modular genes. **(A-C)** Results of GO analysis of 27 genes, biological process (BP), cellular component (CC) and molecular function (MF) of the genes. **(D)** Results of KEGG analysis of 27 genes.

For biological processes in GO enrichment analysis, biological processes were highly enriched in mitotic cell cycle processes (**Figure 4A**, biological processes (BP)). And for the cellular components in GO, it involves intracellular non-membrane-bound organelles, chromosomes, and mitotic regions (**Figure 4B**, cellular components (CC)). For the molecular functions enriched in GO, including nucleotide binding, phosphoribosylation, chromatin binding (**Figure 4C**, Molecular Functions (MF)). Based on the KEGG database further to decipher the biological pathways behind, the enriched molecular pathways included cell cycle, microRNAs in cancer, oocyte meiosis, breast cancer, gastric cancer, and mTOR signaling pathway (**Figure 4D**). These findings are in line with the results of GO enrichment analysis, providing further evidence of the association between cervical squamous cell carcinoma and psoriasis.

**Figure 5:**
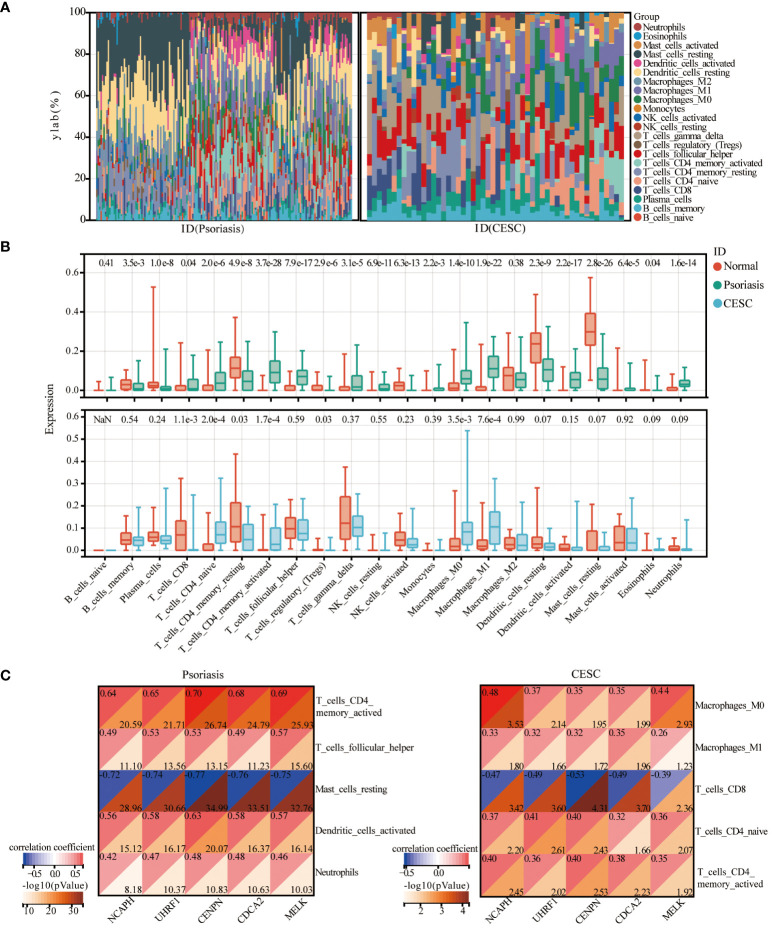
Immune cell infiltration analysis. **(A)** Heat map of the relative proportions of 22 types of infiltrating immune cells in patients with psoriasis and cervical cancer. **(B)** Violin plot of the abundance of each type of immune cell infiltration in the psoriasis and cervical cancer group. **(C)** Correlation graph representing the association of immune cells with five central genes.

The CIBERSORT analysis tool calculated the proportions of 22 types of leukocyte subpopulations in psoriasis and CESC samples, respectively, including naïve B cells, memory B cells, plasma B cells, CD8 T cells, CD4 naive T cells, CD4 memory quiescent T cells, CD4 memory-activated T cells, follicular helper T cells, regulatory T cells (Tregs), γ δ T cells, resting natural killer (NK) cells, activated NK cells, monocytes, M0, M1 and M2 macrophages, resting and activated myeloid dendritic cells, and resting and activated mast cells. We also explore the relationship of key genes to immune infiltrating cells in both diseases and found that genes associated with psoriasis can also play a role in cervical squamous cell carcinoma. Stacked bar graphs of the two datasets show the percentage of 22 immune cells in each sample (**Figure 5A**). Analysis of the immune microenvironment in psoriasis patients revealed significant differences in the abundance of 20 immune cells. Analysis of the immune microenvironment in patients with cervical squamous cell carcinoma revealed notable variations in the abundance of seven immune cells. These differences were statistically significant (**Figure 5B**). In summary, patients with psoriasis and cervical squamous cell carcinoma have varying degrees of multiple immune cell infiltrations, and these immune cell infiltrations may be potential regulatory points for therapy. Then, the spearman correlation coefficient between hub genes and the infiltration level of the immune cell was calculated. As a result, resting mast cells and CD8T cells were negatively correlated with the expression of *NCAPH, UHRF1, CDCA2, CENPN* and *MELK* in patients with psoriasis and cervical squamous carcinoma, respectively (**Figure 5C**).

### Identification of candidate small molecule compounds for the treatment of psoriasis and cervical squamous cell carcinoma

**Figure 6:**
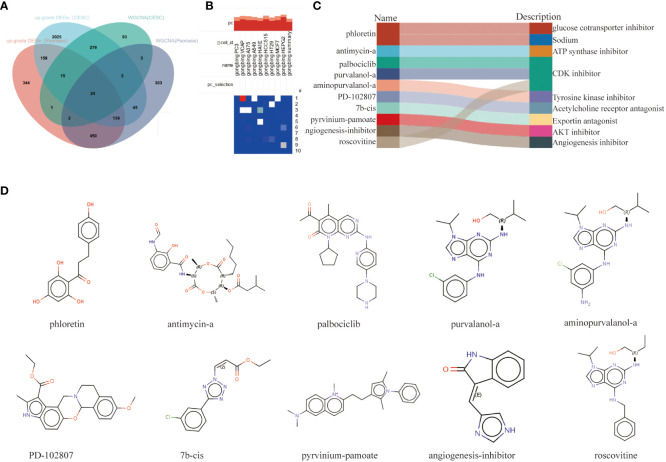
Screening of the potential small-molecular compounds for the treatment of psoriasis and CESC via cMAP analysis. **(A)** Intersection Wayne plots of DEGs genes up-regulated in psoriasis and cervical cancer with hub genes taken from the WCGNA module. **(B)** Heatmap of the top 10 compounds with the highest enrichment in 10 cell lines based on cMAP analysis. **(C)** Top 10 compounds information and targeting pathways. **(D)** Chemical structures of the 10 compounds.

The intersection of DEGs genes upregulated in psoriasis and cervical squamous cell carcinoma was taken with hub genes in the WCGNA module, and 24 relevant pathogenic genes were obtained (**Figure 6A**). The screened 24 relevant pathogenic genes were imported into connectivity map (cMAP) database to predict small molecule compounds that could reverse the gene expression alterations in psoriasis-related pathogenesis and cervical squamous cell carcinoma. Phloretin, antimycin-a, palbociclib, purvalanol-a, aminopurvalanol-a, PD-102807, 7b-cis, pyrvinium-pamoate, angiogenesis-inhibitor, roscovitine were the top 10 compounds with the highest negative scores as potential drugs for therapy (**Figure 6B**). The targeting pathways and chemical structures of these 10 compounds are described in **Figures 6C, D**.

### Validation of hub genes with GEO and TCGA databases and cellular experimental validation

**Figure 7:**
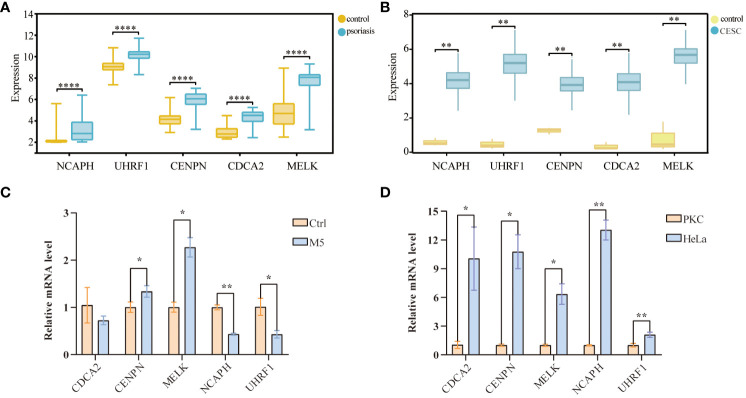
Validation of hub-genes in external datasets and experiments databases. **(A)** Validation of the center gene of cervical cancer in TCGA-CESC database. **(B)** Validation of center gene in the psoriasis dataset GSE35182. **(C)** RT-qPCR results of 5 key genes in psoriasis cell samples. **(D)** RT-qPCR results of 5 key genes in cervical cancer cell samples. (*p< 0.05, **p < 0.01, ***p < 0.001).

To further confirm the accuracy of the comprehensive bioinformatics analysis described above, we first examined the expression patterns of the five hub genes in the patients of the two validation cohorts, and chose the psoriasis dataset, GSE63514 and the cervical squamous cell carcinoma dataset, TCGA-CESC, as the validation datasets. Multi-group box plots showed that the expression levels of *NCAPH, UHRF1, CDCA2, CENPN* and *MELK* were significantly higher in psoriasis patients and cervical squamous cell carcinoma patients than in normal controls (**Figures 7A, B**). RT-qPCR results confirmed that the expression levels of *CENPN* and *MELK* mRNA levels were increased (**Figure 7C**), and that the expression of the five pivotal genes were consistently up-regulated in cervical cancer samples as compared to the control samples (**Figure 7D**).

### Cohort validation of hub genes and enrichment analysis

**Figure 8:**
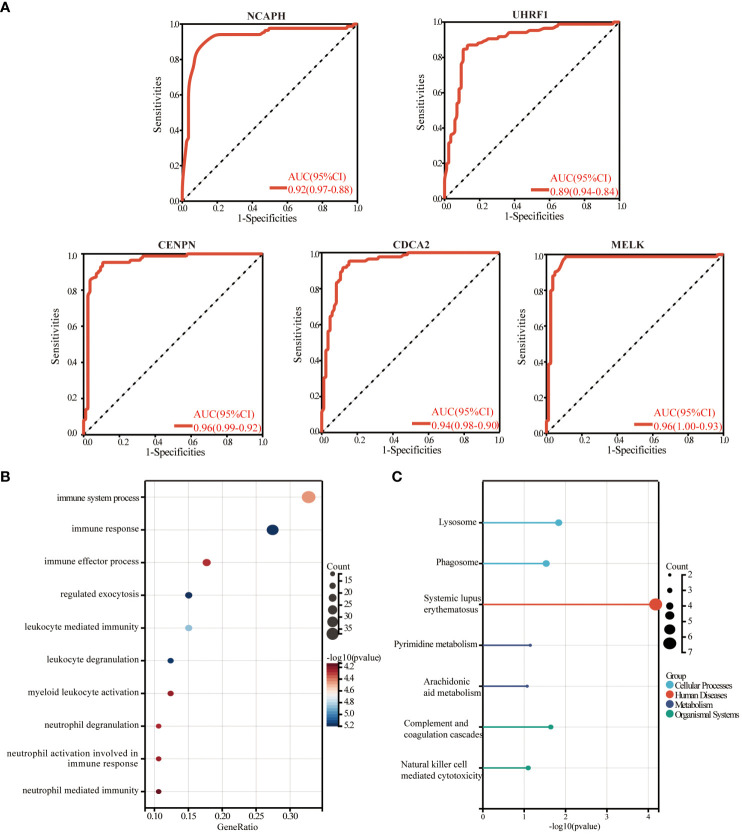
The diagnostic value evaluation in the validation cohort and enrichment analysis. **(A)** ROC plot of each key gene (*NCAPH, UHRF1, CDCA2, CENPN*, and *MELK*) based on the AUC. **(B)** The bubble plot demonstrates the results of GO enrichment analysis of hub gene-related differential genes in psoriasis. **(C)** The results of the KEGG enrichment analysis of hub gene-related differential genes in psoriasis are demonstrated by a lollipop plot. AUC, area under the curve.

We plotted ROC curves based on the five candidate genes to assess the diagnostic value of each gene. The calculated AUCs and 95% confidence intervals were as follows: *NCAPH* (AUC 0.92, CI 0.97–0.88), *UHRF1* (AUC 0.89, CI 0.94–0.84), *CDCA2* (AUC 0.96, CI 0.99–0.92), *CENPN* (AUC 0.94, CI 0.98–0.90) and *MELK* (AUC 0.96, CI 1.00–0.93). The findings indicated that the acquired genes had a significant diagnostic value in Psoriasis (**Figure 8A**). To investigate the potential functions of common central genes, we divided the samples from the psoriasis dataset into groups with high and low expressions based on median levels. We then identified DEGs between these groups and conducted GO/KEGG enrichment analysis. The significant enriched genes include “lysosomes, phagocytosis, SLE, pyrimidine metabolism, arachidonic acid metabolism, complement and coagulation cascades, and natural killer cell-mediated cytotoxicity (**Figures 8B, C**).

### The regulatory signatures analysis

**Figure 9:**
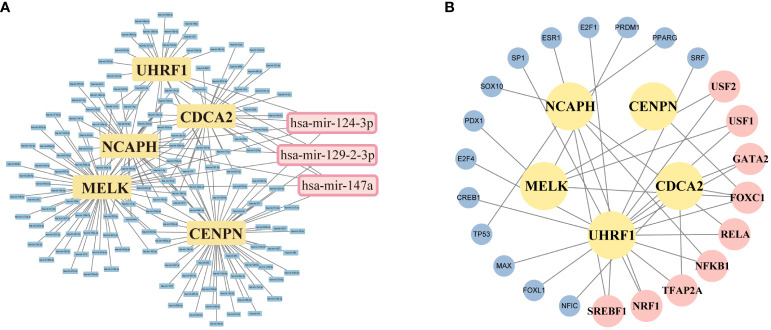
Screening of potential miRNAs and TF-mRNA network of 5 targeting hub-gene. **(A)** An Interaction network of five hub genes and potential miRNAs-targeted. **(B)** TF-mRNA network of 5 hub genes. The pink squares represent the top TFs associated with the hub genes.

We applied the miRNet database to screen the targeted miRNAs of NCA *NCAPH, UHRF1, CDCA2, CENPN* and *MELK*. As depicted in **Figure 9A**, the prediction identifies three miRNAs: hsa-miR-124–3p, hsa-mir-129–2-3p, and hsa-mir-147a. The Network analysis tool explored 9 transcription factors namely FOXC1, NFKB1, RELA, SREBF1, NRF1, GATA2, TFAP2A, USF1, USF2 (**Figure 9B**). The TFs and miRNAs related to three hub genes via network analysis were shown in **Table 4**.

**Table 4:** Top transcription factors and miRNA predicted from miRNA-mRNA, TFs-mRNA regulatory networks.

TFs/miRNAs	Description	Biological function	Reference
FOXC1	Forkhead	Regulation of cell proliferation, migration and invasion through PI3K/AKT signaling	(46)
NFKB1	nuclear factor kappa B subunit 1	Inhibition of cell proliferation, colony formation and migration in cervical cancer	(47)
RELA	v-rel avian reticuloendotheliosis viral oncogene homolog A	Control of NF-κB activity by autophosphorylation in inflammatory diseases and cancer。	(48)
SREBF1	sterol regulatory element binding transcription factor 1	Stimulates ubiquitination of SREBP1 and inhibits endoplasmic reticulum stress in CESC cells.	(49)
NRF1	nuclear respiratory factor 1	Leads to severe oxidative stress, genomic instability	(50)
GATA2	GATA binding protein 2b	A common regulatory elements in cervical cancer	(51)
TFAP2A	transcription factor AP-2 alpha	Promotes the growth of cervical tumors	(52, 53)
USF1/2	upstream transcription factor 1/2	Enhancement of cervical cancer cell malignancy by transcriptional activation of p65	(54, 55)
hsa-miR-124–3p	MicroRNA 124	Direct targeting of IGF2BP to inhibit cervical cancer growth and metastasis is considered to be an important marker and target for CC prognosis	(56)
hsa-mir-129–2-3p	MicroRNA 129	The methylation process of mir-129–2-3p increases cervical (pre)cancerous lesions.	(57)
hsa-mir-147a	MicroRNA 147a	Interacts with circ_0018289 binding and Linc00319 to promote cervical cancer progression.	(58, 59)

## Discussion

Cervical cancer is the fourth leading cause in cancer incidence and mortality among women, contributing to over 60,000 new cases and approximately 342,000 deaths across the world (60). In recent years, there has been a decline in the incidence of cervical cancer due to high-risk group screenings. Despite some progress, the 5-year survival rate for patients with advanced cervical cancer is only 16.7%. And early recognition and diagnosis of cervical cancer is one of the best measures to improve prognosis and reduce social burden (61).

Psoriasis, a chronic inflammatory skin disease, is increasingly recognized as a systemic inflammatory condition and can coexist with other diseases (62). The link between psoriasis and cancer is also gaining attention. In a cohort study, individuals who underwent treatment for severe psoriasis displayed a 41% greater likelihood of succumbing to malignant tumors than non-psoriasis attendees (63). A meta-analysis of 11 retrospective studies showed an increased risk of cancer in non-melanoma skin cancer (NMSC) (95% confidence interval [CI] 1.07–1.25) (64). A cohort study assessing cancer risk among psoriasis patients in the United Kingdom also found an increased risk of NMSC, lung cancer, and lymphoma, and this study also removed the effects of confounding factors such as smoking and alcohol consumption (65). Specifically cervical cancer, surveys have demonstrated that psoriasis patients taking biologics were more likely to be screened for cervical cancer than the general population without psoriasis (adjusted hazard ratio [HR] 1.09; 95% [CI] 1.02 - 1.16) (66). In addition, psoriasis lesions have been shown to contain HPV infection (67). Due to the immunomodulatory effects of medications used to treat psoriasis, which contribute to the development of cervical cancer, the ability of clearing HPV infection is impaired, leading to an increased risk of cervical tumors. This suggests that patients with psoriasis are at increased risk of developing HPV-associated cervical lesions; there may be a co-morbid mechanism and risk association between the two, and our study provides new insights for clinicians to be aware of encouraging patients with psoriasis to follow a cervical tumor screening program (68).

Combining WGCNA, limma difference analysis and machine learning, we screened five key genes as markers of psoriasis and cervical cancer co-morbidities, including *NCAPH, UHRF1, CDCA2, CENPN* and *MELK*. *NCAPH* predominantly promotes sister chromatid entanglement, exacerbating chromosome segregation errors and cell division failure (69). Studies have confirmed that elevated levels of NCAPH expression are associated with an unfavorable prognosis and immune infiltration in several cancer types, including lung adenocarcinoma, breast cancer, and colorectal cancer (70). The expression of *NCAPH* in cervical cancer tissues was significantly higher than that in normal cervical tissues and was significantly correlated with the size, invasion and lymph node metastasis of cervical cancer tumor tissues, suggesting that *NCAPH* is a potential target for cervical cancer immunotherapy (71). *UHRF1* is a highly expressed epigenetic regulator within cancer cells that plays a significant role in double-strand break repair through homologous recombination. Overexpression of UHRF1 results in increased DNA methylation, promoting the further development, progression, and invasion of cancer (72, 73). Interestingly, human papillomavirus was found to induce cervical cancer through *UHRF1*-mediated promoter methylation, suggesting that treatment targeting *UHRF1* may inhibit cervical carcinogenesis through cell cycle arrest and apoptosis (74–77). The mitochondrial protein CENP-N regulates normal chromosome segregation by recognizing histone H3 in filamentous nucleosomes and promoting densification of filamentous chromatin (78, 79). In this study, *CENPN* expression was significantly elevated in both psoriasis and cervical cancer tissues compared to control samples, which could serve as a potential diagnostic indicator for identifying cervical cancer in psoriasis patients. In conclusion, our study suggests that these five central genes may play a key role in psoriasis and cervical cancer.

The pathophysiology of psoriasis involves abnormal activation of the autoimmune system, both intrinsic and acquired. This dysregulation is a key component of mechanisms that prevent and interfere with cancer (79). There exists a robust association between cancer and inflammation, with inflammation representing a paramount risk factor in the development of cancer, often accompanied by inflammation (80). We explored the mechanisms of immune dialog between psoriasis and cervical cancer. Our study demonstrated that cervical cancer tissues are heavily infiltrated with T lymphocytes and the ratio of CD4+ to CD8+ is reversed, and there is evidence that this phenomenon promotes an inflammatory response in patients with cervical cancer, leading to elevated levels of CRP(C-reactive protein) and HbA1c% (81). Interestingly, previous studies have shown that Th1 subpopulation T cells promote macrophage- and cytotoxic T cell-mediated immune responses through the release of interferon-γ (IFN-γ) and TNF-α, which are key factors in the pathogenesis of psoriasis (82). In addition, our immune infiltration analysis showed that macrophage type M1, which promotes the development of inflammation, was also heavily infiltrated in cervical cancer tissues. It has been shown that depletion of macrophages attenuates psoriatic inflammation and reduces the levels of Th1 cytokines, including IL-1α, IL-6, IL-23, and TNF-α, to normal levels (83–86). Psoriasis and cervical cancer show common properties and potential in terms of immune processes.

Although biologics have shown better efficacy in psoriasis, the side effects of biologics pose certain hazards. Therefore, there is an urgent need to explore potential drugs. Small molecule compounds have the advantages of high tissue permeability, adjustable half-life, and high oral bioavailability, resulting in better therapeutic efficacy. We linked causative genes associated with psoriasis and cervical cancer through cMAP analysis to identify potential therapeutic agents. roscovitine, palbociclib, and purvalanol-a are CDK (cell cycle protein-dependent kinase) inhibitors. The CDK inhibitors block the proliferation inhibition of malignant tumor cells through cell cycle progression (87). In some inflammation models, roscovitine demonstrates a reduction in leukocyte-mediated inflammation (88). Pravachol A, a CDK2 inhibitor, induces apoptosis in human neutrophils (89). Most solid tumor cells produce energy by relying heavily on aerobic glycolysis, and phloretin can effectively inhibit cancer progression by targeting the glycolytic pathway as a glucose cotransporter inhibitor (90). Antimycin A is a promising anticancer agent (90), which can target mitochondria, reduce human papillomavirus E6/E7 oncogene protein, inhibit proliferation, and induce apoptosis in cervical cancer cells (91). Aminopurinol A as a Tyrosine kinase inhibitor can restore the abnormal process of pre-mRNA splicing in cancer (92). The anticancer effects of Pyrviniu are mainly manifested in the inhibition of mitochondrial function as well as the renewal of cancer stem cells (93), and in particular, it significantly impedes cancer cell invasion via the Wnt/β-catenin signaling pathway (94). These drugs have promising potential in the treatment of psoriasis and cervical cancer.

We recognize the potential challenges faced by patients with comorbidities. For example, the use of biologics during treatment tends to suppress the activation of the body's immune system, which implies an increased potential risk of tumorigenesis. To further validate this concern in patients with psoriasis treated with biologics, we need to conduct additional clinical cohort studies. How psoriasis and cervical cancer talk through key genes under the systemic neuro-immune-endocrine network also needs further experimental exploration.

## Conclusion

Based on bioinformatics analysis and machine learning, we systematically identified five related candidate genes (NCAPH, UHRF1, CDCA2, CENPN and MELK). This study will facilitate the exploration of molecular mechanisms, particularly with regard to the immune response and drug action. A comprehensive understanding of disease pathogenes is vital for mediating their interaction and prevent the risk of complications. The screened genes could be used for clinical diagnosis and treatment.

## Data availability statement

The original contributions presented in the study are included in the article/supplementary material. Further inquiries can be directed to the corresponding authors.

## Ethics statement

The studies involving humans were approved by Ethics Committee of Xi‘an Jiaotong University. The studies were conducted in accordance with the local legislation and institutional requirements (No.2022-1012). The participants provided their written informed consent to participate in this study.

## Author contributions

LL: Conceptualization, Methodology, Validation, Writing – original draft, Writing – review & editing. PY: Formal analysis, Investigation, Supervision, Writing – original draft, Writing – review & editing. RY: Data curation, Project administration, Software, Writing – original draft, Writing – review & editing. GZ: Funding acquisition, Resources, Visualization, Writing – review & editing. CW: Conceptualization, Investigation, Software, Writing – review & editing. YZ: Data curation, Funding acquisition, Methodology, Supervision, Writing – review & editing. SW: Funding acquisition, Validation, Visualization, Writing – review & editing. ML: Funding acquisition, Methodology, Project administration, Resources, Supervision, Validation, Writing – review & editing.
